# Assessing trends and predictors of tuberculosis in Taiwan

**DOI:** 10.1186/1471-2458-12-29

**Published:** 2012-01-12

**Authors:** Chung-Min Liao, Nan-Hung Hsieh, Tang-Luen Huang, Yi-Hsien Cheng, Yi-Jun Lin, Chia-Pin Chio, Szu-Chieh Chen, Min-Pei Ling

**Affiliations:** 1 Department of Bioenvironmental Systems Engineering, National Taiwan University, Taipei, Taiwan 10617, Republic of China; 2 Department of Public Health, Chung Shan Medical University, Taichung, Taiwan 40201, Republic of China; 3 Department of Family and Community Medicine, Chung Shan Medical University Hospital, Taichung, Taiwan 40242, Republic of China; 4 Department of Health Risk Management, China Medical University, Taichung, Taiwan 40201, Republic of China

**Keywords:** Tuberculosis, Seasonality, Weather, Aboriginal health, Poisson regression model, Taiwan

## Abstract

**Background:**

Variety of environmental and individual factors can cause tuberculosis (TB) incidence change. The purpose of this study was to assess the characteristics of TB trends in the period 2004 - 2008 in Taiwan by month, year, gender, age, temperature, seasonality, and aborigines.

**Methods:**

The generalized regression models were used to examine the potential predictors for the monthly TB incidence in regional and national scales.

**Results:**

We found that (*i*) in Taiwan the average TB incidence was 68 per 100,000 population with mortality rate of 0.036 person^-1 ^yr^-1^, (*ii*) the highest TB incidence rate was found in eastern Taiwan (116 per 100,000 population) with the largest proportion of TB relapse cases (8.17%), (*iii*) seasonality, aborigines, gender, and age had a consistent and dominant role in constructing TB incidence patterns in Taiwan, and (*iv*) gender, time trend, and 2-month lag maximum temperature showed strong association with TB trends in aboriginal subpopulations.

**Conclusions:**

The proposed Poisson regression model is capable of forecasting patterns of TB incidence at regional and national scales. This study suggested that assessment of TB trends in eastern Taiwan presents an important opportunity for understanding the time-series dynamics and control of TB infections, given that this is the typical host demography in regions where these infections remain major public health problems.

## Background

Tuberculosis (TB) is caused by infection with the pathogen *Mycobacterium tuberculosis *[1]. A recent World Health Organization (WHO) report documented the diagnosis of nearly 10 million new cases of TB in 2007 with an estimated 1.3 million deaths from TB in the same year [2]. Therefore, TB remains a leading cause of death resulting in high morbidity and mortality worldwide, with an estimate of one-third of world's population is infected with TB bacilli [2]. On the basis of these statistics, TB is among the top ten causes of death worldwide. Despite predictions of a decline in global incidence, the number of new cases is continuous to grow.

Over 50% of global TB cases are found in Southeastern Asia and the Western Pacific [2]. The TB incidence and mortality rates in Taiwan varied geographically, with higher rates in southern and eastern regions than in the northern region [3]. The epidemiological study in Taiwan found that incidence and mortality rate of TB infection were 62.0-74.6 (per 100,000 population) and 3.3-5.7 (per 100,000 per population) in the period 2002-2008, respectively [4]. The notification rate and mortality rate were higher in eastern Taiwan than in the national scale [5]. Hsueh et al. [3] found that aborigines and people living in mountainous regions in eastern Taiwan had higher incidence rates.

Hsueh et al. [3] and Lee et al. [4] implicated that high disease burden of TB and inadequate current control infrastructure and training for TB implementation, e.g., directly observed treatment short-course (DOTS) strategy, are posing a great impact on public health in Taiwan, leading to current challenges to TB control such as the increasing burden of patients with multidrug-resistant TB infection, the persistent high rate of mortality, and unsatisfactory compliance of treatment.

Previous studies have reported a strong association between seasonality and TB in temperate, tropical, and subtropical regions [6-13]. They found that seasonal peaks of TB cases generally occurred at the end of winter [9,11] and at the beginning of the summer season [7,8,10]. They implicated that increased indoor exposure in winter, diagnostic delays, and an association with vitamin D levels explained this phenomenon [6,14]. There are strong interactions between the effects of seasonality and the effects of weather because seasonality might influence the season of emergence of active TB, making both sensitive to additional stresses such as climate change. Little is known about the time-series dynamics of TB trends in Taiwan taking into account seasonal patterns and weather effects.

Dye and Williams [15] have characterized the impact of key demographic and epidemiological factors on TB trends including (*i*) epidemiological differences among populations because some infected people are at high risk of developing active TB and some kinds of people and patients transmit more infections than others, (*ii*) demographic transition because populations are aging and a relatively high risk of TB among the elderly is a real possibility, and (*iii*) rejuvenation of the TB epidemic among young adults.

It is believed that by understanding the factors that affect TB trends will help to make control programs more successful. Despite this far-reaching importance, empirical estimates of long-term trends in TB epidemics in Taiwan remain limited. On the other hand, despite differences in scale and approach, it is clear that long-term estimates of TB trends are a necessary. The purpose of this study was to assess the characteristics of TB trends from 2004-2008 in Taiwan by month, year, gender, age, temperature, seasonality, aborigines/nonaborigines and to provide pronounced generalized regression models to examine the potential predictors for the monthly TB incidence in regional and national scales.

## Methods

### Background information

Typically, in subtropical Taiwan (22°-25°N and 120°-122°E), the average (± standard deviation) temperatures during spring (March to May), summer (June to August), fall (September to November), and winter (December to February) from 1999 to 2009 were 22.27 ± 2.97, 29.07 ± 1.00, 24.55 ± 2.96, and 17.37 ± 1.17°C, respectively, with a relative humidity ranging from 75-78% and a maximum rainfall occurring in June (7800 mm) and August (8527 mm) (Taiwan Central Weather Bureau, http://www.cwb.gov.tw/V7/index.htm). Typically, most tropical and subtropical countries experience these weather conditions.

### Study data

Monthly-based disease burden of TB data by gender, age groups, and subpopulation groups in Taiwan were obtained from reports of Centers for Disease Control of Taiwan (Taiwan CDC) for the period 2004-2008 [4]. According to Taiwan CDC (http://www.cdc.gov.tw) [16], we geographically divided Taiwan into six regions of northern, central, southern, southern Kao-Ping, eastern Hwa-Tung, and island regions to analyze the trends of TB incidence. We calculated the TB incidence rate (per 100,000 population) by annual regional confirmed TB case over total regional population number. We also pooled all regional data to represent national scale of TB cases. Here we used mean age and ratio of male and female TB cases as the input data. Note that we calculated the prevalence of TB from the product of incidence and duration of illness. The average monthly mean, maximum, and minimum temperature data were obtained from Taiwan Central Weather Bureau in the period 2004 to 2008. We also compiled the subpopulation of aborigine data in local region of Taiwan. There were nearly 79,000 and 89,000 aborigines in Taitung and Hwalien County, respectively, and were approximately 34% of total aborigine population in Taiwan (MOI, http://www.moi.gov.tw/stat/index.aspx) [17]. In addition, Hwalien County is the major region having the largest aborigines' population. All data used in this research are openly available.

### Statistical analyses

Spearman rank correlation tests were performed to investigate the correlation between TB incidence rates and minimum, mean, and maximum temperatures and further to examine the lagged effects with a lag of 0 to 4 months of temperature on TB incidence. TB trends were estimated for two selected regions where the highest TB incidence was evident among six study areas in the period 2004-2008.

Given the evidence for the roles of seasonality, temperature, age, and gender as potential predictors of TB trends, we used a Poisson regression model to assess the characteristics of TB epidemic in Taiwan in the period 2004-2008. The model was fitted to the blended data to estimate TB trends as follows,

(1) $$Y\left(t\right)=\exp\left(\begin{array}{c} \beta_{0}+\beta_{1}t+\beta_{2}t^{2}+\beta_{3}\overset{5}{\underset{n=1}{\sum }}\sin\left(\frac{2n\pi t}{12}\right)+\beta_{4}\overset{5}{\underset{n=1}{\sum }}\text{cos}\left(\frac{2n\pi t}{12}\right)+\beta_{5}T_{t-n}\\ +\beta_{6}age_{t}+\beta_{7}male_{t}+\beta_{8}female_{t} \end{array}\right)$$

where *Y*(*t*) is the expected TB incidence at time *t, β*_0 _is the intercept, *t *and *t*^2 ^are the linear and quadratic time trends, respectively, *β*_1 _through *β*_8 _represent the fitted coefficients, $\overset{5}{\underset{n=1}{\sum }}\sin\left(2n\pi t∕12\right)$and $\overset{5}{\underset{n=1}{\sum }}\cos\left(2n\pi t∕12\right)$represent the seasonality with five cycles [18], *T*_t-n _represents the monthly temperatures with *n*-month lag (°C), respectively, *age*_t_, *male_t_*, and *female_t _*represent the monthly TB incidence data based on age, male, and female at time *t*, respectively.

Eq. (1) is one simple expression of generalized linear models that do not require prior knowledge of the shape of the response function. To assess the characteristics of TB trends of aborigine subpopulations, the Poisson regression model in Eq. (1) was also fitted with the available TB data for the selected study regions in question. To ensure robustness, the model was tested by forecasting dynamic TB time-series in 2008 based on TB data in the period 2004-2007.

The Statistica^® ^software (Version 6.0, StatSoft, Tulsa, OK, USA) was used to perform Spearman's rank correlation tests and other related statistical analyses. Poisson regression model was conducted in the open-source language R (Version 2.11.1, The R Foundation for Statistical Computing, 2010). To compare modeled and observed results, the best fit was evaluated using root-mean-squared-error (RMSE), computed from $\text{RMSE}=\sqrt{\sum_{n=1}^{N}{\left(I_{m,n}-I_{s,n}\right)}^{2}/N}$ where *N *denotes the number of measurements, *I_m,n _*is the TB incidence data, and *I_s,n _*is the simulation result corresponding to data point *n*. Akaike information criterion (AIC) was also used to assess model fit and can be expressed as AIC = 2 *k*-2ln(L) where *k *is the number of predictors in the Poisson regression model, and *L *is the maximized value of the likelihood function for the estimated model. The minimum of RMSE and AIC can be used to judge the model fitness in data description and prediction.

## Results

### Quantitative TB epidemiological data

Table 1 summarizes the estimates of TB incidence, prevalence, mortality rate, and relapse proportion for major counties and cities situated at northern, central, southern, and eastern Taiwan, respectively, for the period 2004-2008. We found that the incidence rates were higher in Pingtong County at southern Taiwan (108 per 100,000 population) and Taitung (104 per 100,000 population) and Hwalien (124 per 100,000 population) Counties at eastern Taiwan. Taipei City of northern Taiwan, however, had lower average incidence/prevalence/mortality rates and relapse proportion with an incidence of 50 per 100,000 population.

**Table 1:** Incidence, prevalence, relapse and mortality rates of TB in Taiwan region (2004-2008)

Area/county/city	Incidence Rate^a^	Prevalence Rate^b^	Relapse Proportion^c^	TB mortality Rate^d^
	
	Mean (SD)	Mean (SD)	Mean (SD)	Mean (SD)
Taiwan	68 (5)	88 (5)	4.38 (0.11)	0.036 (0.013)

Northern Taiwan	57 (3)	74 (3)	3.77 (0.12)	0.032 (0.01)

Ilan County	78 (2)	99 (2)	5.04 (0.86)	0.031 (0.015)

Taipei City	50 (4)	65 (3)	2.70 (0.26)	0.033 (0.013)

Taipei County	60 (23)	79 (3)	4.23 (0.51)	0.028 (0.009)

Keelung City	80 (4)	104 (6)	4.01 (0.61)	0.025 (0.003)

Taoyuan County	57 (2)	74 (2)	4.13 (0.32)	0.033 (0.016)

Hsinchu County	53 (4)	69 (6)	2.75 (0.30)	0.034 (0.009)

Hsinchu City	48 (2)	62 (2)	1.30 (0.21)	0.041 (0.018)

Miaoli County	51 (8)	66 (10)	4.43 (0.23)	0.050 (0.023)

Central Taiwan	68 (4)	88 (6)	4.32 (0.20)	0.040 (0.015)

Taichung County	58 (4)	75 (5)	3.69 (0.44)	0.035 (0.023)

Taichung City	54 (2)	70 (2)	4.52 (0.30)	0.040 (0.019)

Changhwa County	73 (3)	95 (4)	3.87 (0.15)	0.057 (0.019)

Nantou County	86 (9)	112 (11)	5.64 (0.24)	0.034 (0.015)

Yunlin County	87 (10)	113 (13)	3.89 (0.53)	0.027 (0.005)

Chiayi County	74 (8)	97 (10)	4.90 (1.48)	0.036 (0.021)

Chiayi City	55 (5)	72 (7)	6.26 (1.23)	0.028 (0.010)

Southern Taiwan	83 (6)	108 (8)	4.67 (0.05)	0.038 (0.015)

Tainan County	74 (5)	96 (6)	4.07 (0.02)	0.031 (0.013)

Tainan City	55 (4)	72 (5)	6.08 (0.08)	0.029 (0.013)

Kaohsiung City	78 (5)	102 (7)	4.27 (0.26)	0.041 (0.015)

Kaohsiung County	96 (8)	125 (10)	5.11 (0.04)	0.034 (0.015)

Pingtung County	108 (10)	140 (12)	4.53 (0.09)	0.052 (0.021)

Eastern Taiwan	116 (10)	151 (13)	8.17 (0.54)	0.040 (0.019)

Taitung County	104 (10)	136 (13)	7.29 (1.61)	0.045 (0.026)

Hwalien County	124 (10)	162 (13)	8.68 (0.07)	0.037 (0.015)

^a ^Incidence rate (per 100,000 population) (2004-2008): Annual regional confirmed TB case/total regional population number [4].

^b ^Prevalence rate (per 100,000 population): Annual regional incidence rate × duration of illness (= 1.3 year) based on Taiwan Tuberculosis Control report (2007-2009) [30].

^c ^Relapse proportion (2007-2008): (Annual regionally relapsed case/total regionally notification case) × 100. Adopted from Taiwan CDC database and Taiwan Tuberculosis Control report [30].

^d ^TB mortality rate (person^-1 ^yr^-1^) (2004-2008): Annual regionally TB induced mortality/annual regional incidence [4].

Overall, in Taiwan the average TB incidence, mortality, and relapse proportion were estimated to be 68 per 100,000 population, 0.036 person^-1 ^yr^-1^, and 4.38%, respectively, for the period 2004-2008 (Table 1). The highest TB incidence rate was found in eastern Taiwan (116 per 100,000 population) with the estimated prevalence, mortality rate, and relapse proportion of 151 per 100,000 population, 0.040 person^-1 ^yr^-1^, and 8.17%, respectively, for the period 2004-2008 (Table 1). The eastern Taiwan had the largest proportion of TB relapse cases (8.17%) in which Hwalien County gave 8.68% relapse proportion (Table 1).

### Regional and national TB trends

**Figure 1:**
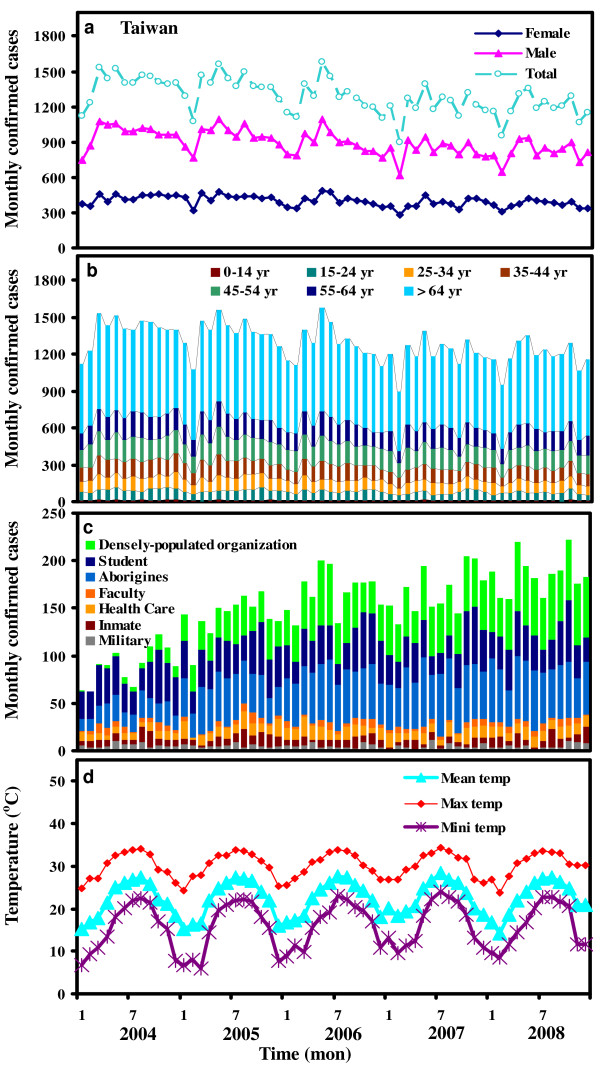
**Time-series dynamics of (**a**) gender-, (**b**) age group-, and (**c**) subpopulation-specific monthly confirmed TB cases in Taiwan in the period 2004-2008**. The densely-populated organization is defined as the people living and working in long-term care center and nursing home for children and elderly. The faculty is defined as the people teaching and working in the school and college. (**d**) Time series of monthly mean, maximum, and minimum temperatures in Taiwan in the period 2004-2008.

**Figure 2:**
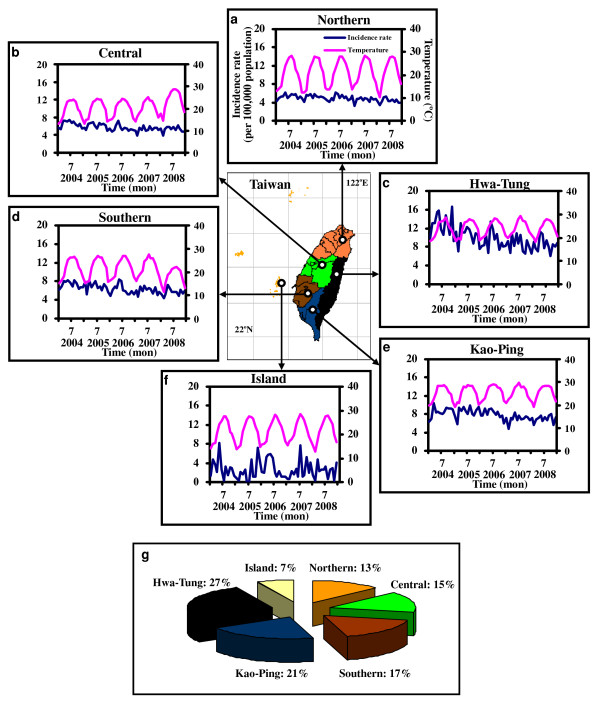
**Time series of TB incidence rate with mean temperature in (**a**) northern, (**b**) central, (**c**) eastern Hwa-Tung, (**d**) southern, (**e**) southern Kao-Ping, and (**f**) island regions, respectively**. (**g**) Region-specific contributions of TB incidence in Taiwan.

Figure 1 demonstrates the time-series dynamics of gender-, age group-, and subpopulation-specific monthly confirmed TB cases together with mean, maximum, and minimum temperatures in Taiwan region in the period 2004-2008. The time-series of TB incidence rate with mean temperature in northern, central, southern, southern Kao-Ping, eastern Hwa-Tung, and island regions were illustrated in Figure 2 Our result indicates that Hwa-Tung region contributed nearly 27% of TB incidence in Taiwan in the period 2004-2008 (Figure 2g). We thus chose Hwalien and Taitung Counties as our study areas compared to Taiwan.

We first estimated the impact of time-lag effect of temperature on TB incidence in Hwalien and Taitung Counties (Table 2). Results indicate that mean temperature with a 3-month lag was highly significant in Hwalien County (Spearman's *ρ *= 0.37; *p *< 0.005). Yet, no significance was found between temperature and TB incidence in Taitung County. Alternatively, we chose maximum temperature with 2-month lag (maximum Spearman's *ρ *= 0.22; *p *= 0.09) to explain the temperature impact on TB incidence in Taitung County. In Taiwan, the mean temperature with 1-month lag had significant effect on TB trends.

**Table 2:** Spearman's rank correlation coefficient (*ρ*) for time-lag effect with temperature (°C) in Taiwan, Hwalien and Taitung Counties, respectively

Time-lag (mon)	Mean temperature	Mini temperature	Max temperature
		**Taiwan**	

0	0.35*	0.28*	0.40**

1	0.42***	0.36**	0.40**

2	0.40**	0.36**	0.38*

3	0.19	0.20	0.17

4	-0.01	-0.02	-0.03

		Hwalien County	

0	0.05	0.05	0.14

1	0.21	0.15	0.23

2	0.30*	0.26*	0.32*

3	0.37**	0.34*	0.35*

4	0.20	0.17	0.19

		Taitung County	

0	0.15	0.13	0.07

1	0.21	0.08	0.15

2	0.21	0.11	0.22

3	0.10	0.07	0.01

4	-0.06	-0.16	-0.10

*p <0.05; **p < 0.005; ***p < 0.001.

**Figure 3:**
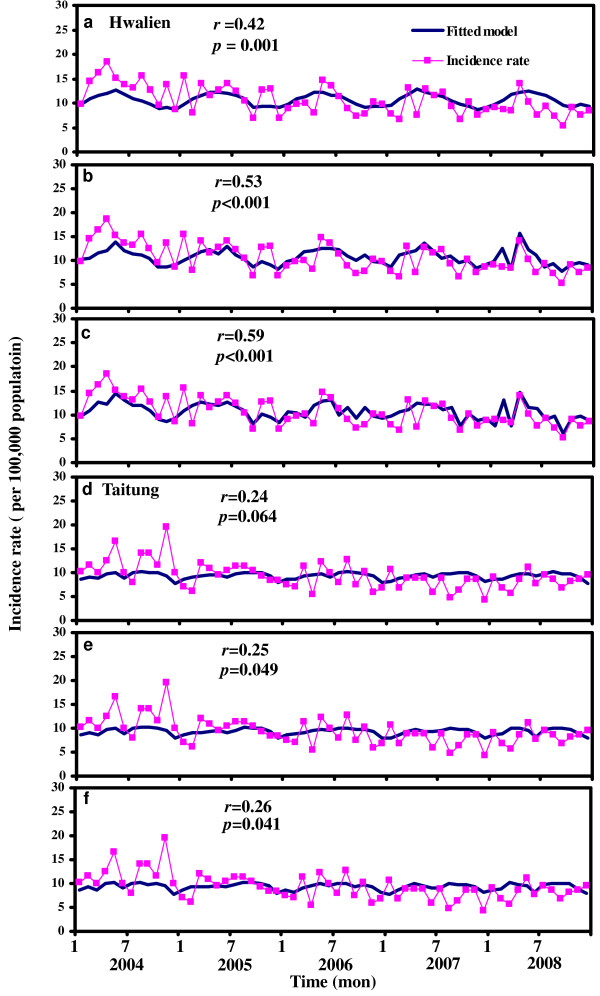
**Fitting of Poisson regression models with lagged temperature for (**a**) Hwalien and (**d**) Taitung**. Fitted models with lagged temperature and age group for (**b**) Hwalien and (**e**) Taitung. Fitted models with lagged temperature, age group and gender for (**c**) Hwalien and (**f**) Taitung in the period 2004-2008. (See Table 3 for detailed models).

Poisson regression models with five cycles of seasonality with the effects of temperature, age group, and gender were used to capture the TB trends estimation, allowing both the quantitative (magnitude) and qualitative (shape) of trends to be estimated (Table 3, Figure 3). Poisson regression model (Eq. (T3)) can estimate TB incidence trends in Hwalien County significantly (*r *= 0.59; *p *< 0.001); whereas the estimation was reasonably well in Taitung County by Poisson model (Eq. (T6)) (*r *= 0.26; *p *< 0.041) (Table 3; Figure 3c, f). Note, however, that only age group (*p *< 0.05) in Hwalien County had a significant effect on TB incidence (AIC = 120.15), whereas 2-month lagged temperature, seasonality, age, and gender all contributed significantly less to TB trends in Taitung County (AIC = 133.37) in the period 2004 - 2008 (Table 4). After performing the Poisson regression model estimation to Taiwan, we found that seasonality, aborigines, male, and age groups all had highly significant effects on TB trends (*p *< 0.001) (Table 5).

**Table 3:** Summary of Poisson regression models used in this study

Poisson regression model^a^	RMSE	
**Hwalien County**		

$Y\left(t\right)=\exp\left[\beta_{0}+\beta_{1}t+\beta_{2}t^{2}+\beta_{3}\overset{5}{\underset{n=1}{\sum }}\sin\left(2n\pi t∕12\right)+\beta_{4}\overset{5}{\underset{n=1}{\sum }}\cos\left(2n\pi t∕12\right)+\beta_{\text{5}}T_{\text{mean},t-3}\right]$	2.66	(T1)

$Y\left(t\right)=\exp\left[\begin{array}{c} \beta_{0}+\beta_{1}t+\beta_{2}t^{2}+\beta_{3}\overset{5}{\underset{n=1}{\sum }}\sin\left(2n\pi t∕12\right)+\beta_{4}\overset{5}{\underset{n=1}{\sum }}\cos\left(2n\pi t∕12\right)+\beta_{5}T_{\text{mean},t-3}\\ +\beta_{6}age_{t} \end{array}\right]$	2.48	(T2)

$Y\left(t\right)=\exp\left[\begin{array}{c} \beta_{0}+\beta_{1}t+\beta_{2}t^{2}+\beta_{3}\overset{5}{\underset{n=1}{\sum }}\sin\left(2n\pi t∕12\right)+\beta_{4}\overset{5}{\underset{n=1}{\sum }}\cos\left(2n\pi t∕12\right)+\beta_{5}T_{\text{mean},t-3}\\ +\beta_{\text{6}}age_{t}+\beta_{7}male_{t}+\beta_{\text{8}}female_{t} \end{array}\right]$	2.34	(T3)

Taitung County		

$Y\left(t\right)=\exp\left[\beta_{0}+\beta_{1}t+\beta_{2}t^{2}+\beta_{3}\overset{5}{\underset{n=1}{\sum }}\sin\left(2n\pi t∕12\right)+\beta_{4}\overset{5}{\underset{n=1}{\sum }}\cos\left(2n\pi t∕12\right)+\beta_{\text{5}}T_{\text{max},t-2}\right]$	2.64	(T4)

$Y\left(t\right)=\exp\left[\begin{array}{c} \beta_{0}+\beta_{1}t+\beta_{2}t^{2}+\beta_{3}\overset{5}{\underset{n=1}{\sum }}\sin\left(2n\pi t∕12\right)+\beta_{4}\overset{5}{\underset{n=1}{\sum }}\cos\left(2n\pi t∕12\right)+\beta_{5}T_{\text{max},t-2}\\ +\beta_{6}age_{t} \end{array}\right]$	2.63	(T5)

$Y\left(t\right)=\exp\left[\begin{array}{c} \beta_{0}+\beta_{1}t+\beta_{2}t^{2}+\beta_{3}\overset{5}{\underset{n=1}{\sum }}\sin\left(2n\pi t∕12\right)+\beta_{4}\overset{5}{\underset{n=1}{\sum }}\cos\left(2n\pi t∕12\right)+\beta_{5}T_{\text{max},t-2}\\ +\beta_{\text{6}}age_{t}+\beta_{7}male_{t}+\beta_{\text{8}}female_{t} \end{array}\right]$	2.63	(T6)

^a^*Y*(*t*) represents expected incidence rate of tuberculosis, *β*_0 _stands for intercept, *β*_1 _stands for linear time trend, *β*_2 _stands for quadratic time trend, *β*_3 _and *β*_4 _stands for the seasonality, *β*_5 _stands for monthly maximum and mean temperature (°C), *t *- 3 in the subscript represents the 3-month lag time and *t *- 2 in the subscript represents the 2-month lag time, *β*_6 _stands for the effect for age, *β*_7 _stands for the effect for male, and *β*_8 _stands for the effect for female.

RMSE = Root mean squared error.

**Table 4:** Fitted coefficients in Poisson regression model for Hwalien and Taitung Counties during 2004-2008

Fitted coefficient		
**Parameter**	**Hwalien County^a^**	**Taitung County^b^**

(Intercept)	3.074	2.287

*T*_mean,t-3_	0.020	

*T*_max,t-2_		-0.012

t	0.074	0.092

*t*^2^	-0.006	-0.007

$\overset{5}{\underset{n=1}{\sum }}\sin\left(2n\pi t∕12\right)$	0.010	-0.006

$\overset{5}{\underset{n=1}{\sum }}\cos\left(2n\pi t∕12\right)$	-0.004	-0.010

Age	-0.010*	0.004

male	-0.194	-0.012

female	-0.724	-0.135

AIC	120.15	133.37

*p < 0.05

^a ^See Table 2 Eq. (T3)

^b ^See Table 2 Eq. (T6)

AIC = Akaike information criterion

**Table 5:** Fitted coefficients in Poisson regression model for Taiwan during 2004-2008

Parameter	Fitted coefficient
(Intercept)	-2.138***

*T*_mean,*t*-1_	-0.008

t	0.147***

*t*^2^	-0.009**

$\overset{5}{\underset{n=1}{\sum }}\sin\left(2n\pi t∕12\right)$	0.042***

$\overset{5}{\underset{n=1}{\sum }}\cos\left(2n\pi t∕12\right)$	-0.014**

Subpopulation	

Non-Aborigine	Ref.^a^

Aborigine	0.700***

Gender	

Female	Ref.

Male	0.749***

Age group	

0-14	Ref.

15-24	2.126***

25-34	2.671***

35-44	3.049***

45-54	3.298***

55-64	3.617***

> 65	4.535***

* *p *< 0.05, ** *p *< 0.005, *** *p *< 0.001.

^a ^Reference category has a value of zero for all the dummy variables.

[Section2, ID = Sec9]

### Aborigine and gender effects on TB incidence

**Figure 4:**
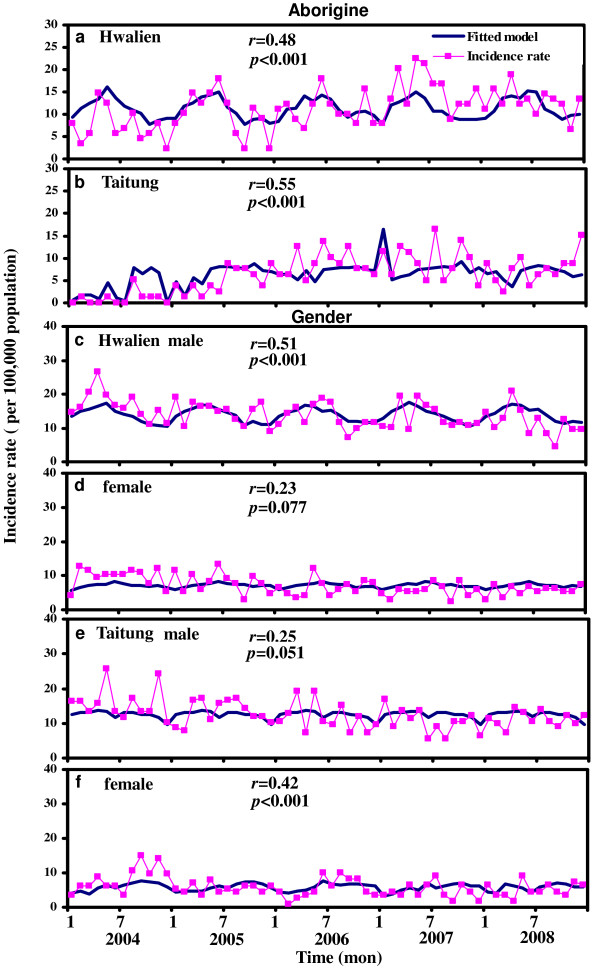
**Comparison of monthly TB incidence rate for aborigine and expected incidence rate for (**a**) Hwalien and (**b**) Taitung based on Poisson regression model**. Comparison of gender-specific monthly TB incidence rate for male and female in Hwalien (**c,d**) and Taitung (**e,f**) in the period 2004-2008.

**Figure 5:**
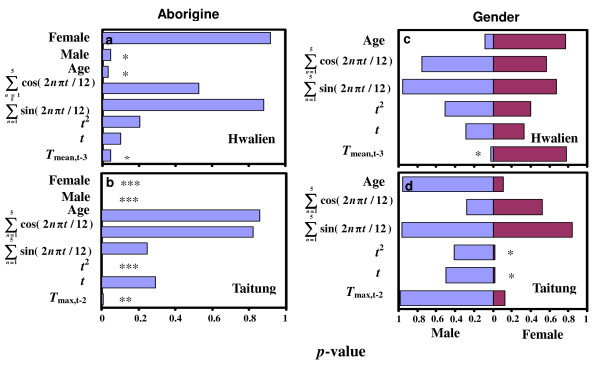
**Significant factors (*p*-value) on TB trends data of aborigines in (**a**) Hwalien and (**b**) Taitung**. Significant factors for gender-specific TB trends data in (**c**) Hwalien and (**d**) Taitung.

To understand the impacts of subpopulation of aborigine and gender on TB trends in Hwalien and Taitung Counties, we fitted Poisson regression models (Eqs. (T3) and (T6); Table 3) to TB incidence data of aborigines and gender, respectively (Figure 44). Results show that Poisson regression models were significantly fitted the aborigine data in Hwalien County (*r *= 0.48; *p *< 0.001) and in Taitung County (*r *= 0.55; *p *< 0.001) (Figure 4a, b). Meanwhile, male had significant effect on TB incidence trends in Hwalien County (*r *= 0.51; *p *< 0.001), whereas TB trends in Taitung County influenced significantly by female effect (*r *= 0.42; *p *< 0.001) (Figure 4c, f). The individual impact of factors on TB trends data of aborigines and gender were illustrated in Figure 5 Significant impacts of gender, time trend, and 2-month lag maximum temperature on aborigine TB trends and only time trends effects on female TB incidence in Taitung County were found (Figure 5b, d).

**Figure 6:**
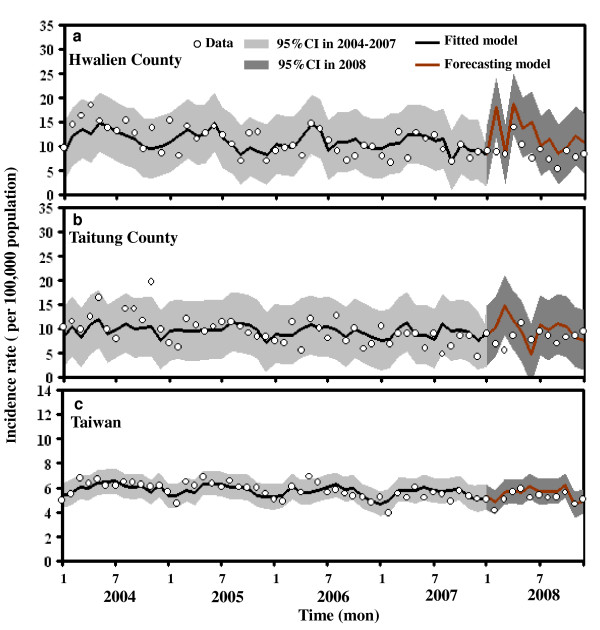
**Forecasted time-series dynamics of TB incidence in 2008 by Poisson regression model based on TB data in the period 2004-2007**.

The fitted Poisson regression models were also tested by forecasting time-series dynamics of TB incidence in 2008 based on TB data from 2004 - 2007. Results show that estimated TB incidence rates followed the proposed regression models in apparent agreement with the observed data in Hwalien County (RMSE = 3.01), Taitung County (RMSE = 3.86), and Taiwan (RMSE = 0.73) during 2008 (Figure 6). In particular, the model accurately predicts relatively similar TB trends in Taiwan during 2008 (Figure 6c).

## Discussion

### TB trends in regional and national scales

This study was to assess the characteristics of TB trends for the period 2004-2008 by month, year, gender, age, temperature, seasonality, aborigines/nonaborigines and to provide a best-fit generalized regression model to examine the potential predictors for TB incidences in Taiwan with regional and national scales. Since TB is caused by infection of the pathogen *M. tuberculosis*, the activity of pathogen can be influenced by weather and environmental conditions. The host behavior and healthy status can also cause the susceptibility of TB infection. Thus we examined different factors which may affect TB events.

We found that Hwalien (124 per 100,000 population) and Taitung (104 per 100,000 population) Counties situated at eastern Taiwan had the highest TB incidences among Taiwan regions in the period 2004-2008. Generally, more than 90% of TB cases found in adults aged 25-64 and older, with the largest fraction among > 64-year olds. The eastern Taiwan had the largest proportion of TB relapse cases (8.17%) in which Hwalien County gave 8.68% relapse proportion in the period 2004-2008. Overall, the average TB incidence, mortality, and relapse proportion were estimated to be 68 per 100,000 population, 0.036 person^-1 ^yr^-1^, and 4.38%, respectively, in Taiwan for the period 2004-2008. Our result indicates that there is a trend of an increasing proportion of TB among the elderly. The plausible reason may be that with the falling of birth rate and increasing of longevity, the aging of populations is rising in Taiwan.

Fares et al., [19] indicated that lower temperature during winter seasons may induce the susceptibility to respiratory epithelium infection. Furthermore, temperature is also an important climatic factor that influences TB seasonal trends. Specifically, the mean temperature with 1-month lag had highly significant effect on TB trends in Taiwan, whereas mean temperature with a 3-month lag showed highly significant in Hwalien County at eastern Taiwan. Therefore, in accordance with the idea of the impact of weather variations on TB infections, temperature emerged as the primary environmental correlate of TB incidence patterns at regional and national scales tested here. Based on this finding, changes in temperature may have strong consequences for the patterns of TB incidence.

There were various reasons that cause temperature lagged effects on TB trends. Naranbat et al., [20] hypothesized that temperature may change the indoor/outdoor (I/O) ratio for TB susceptible and infected population, and further influence the transmission probability for *M. tuberculosis*. Therefore, the longer incubation period may subsequently delay the TB detection and notification. The interval between observable immunological response and infection may delay over 7 weeks [20]. The I/O ratio can also affect Vitamin D intake by sunshine levels which can decrease the risk for TB illness [14,20]. Our results found that there were different lagged effects with temperature on TB incidence such as 1 month-lag in national scale in Taiwan and 3 month-lag in regional scale in Hwalien County. We speculated that climatic properties were the important factor influenced by geographic location, for example, the relative lower temperature and higher rainfall in eastern Taiwan than other regions. The climate factor may further influence the sunshine proportion to residents on Vitamin D intake.

Our findings revealed that there had weak relationship between (lagged) temperature and TB incidence. We thus further considered the seasonality impact on TB incidence separately with temperature effect. The seasonality may exist in many infectious diseases due to variety of human behaviors, environmental conditions, and other uncertain factors. Greenman et al., [21] indicated that external forcing can cause some cycles, oscillations, and even chaotic phenomenon in disease dynamics of populations. Therefore, we considered the seasonality as a major factor which can be seemed as predictor to better understand Taiwan TB trend. The five cycles of seasonality has been applied to investigate periodic trend of monthly new TB cases and possible causes of seasonal trend in previous China TB study [18]. In this study, we also found that the Taiwan TB seasonality had similar pattern with TB epidemic in China. Thus we used five-cycle pattern as seasonality factor to improve the predictability of TB incidence rate. Although the seasonality showed insignificant in regional scales, the properties were detected significantly in national scale.

In regional scale of Hwalien County, age was the only predictor of TB trends identified as statistically significant among considered parameters in the Poisson regression model. Our analysis of TB trends at the regional scale reveals that the appearance of irregular annual TB incidences invariably coincides with the seasonality. None of the considered parameters generally explained variation in TB incidence in Taitung County. Yet, seasonality, aboriginal subpopulation, male, and age groups all showed significant relationships with TB trends in Taiwan. It is interesting to note that gender, time trend, and 2-3-month lag maximum temperature showed stronger association with TB trends in aboriginal subpopulations.

Our study showed that seasonal peaks of TB incidences generally occurred at late spring to early summer seasons in Taiwan. Thus dynamic consequences of seasonal variation in TB incidence appeared in Taiwan. This result was consistent with the study in China [18]. The relatively high significant association of seasonality, gender, age group with national scale TB trends leads to more regular TB time-series dynamics in Taiwan than those in regional Hawlien and Taitung Counties. This emphasizes the potential disadvantages of extrapolating TB trends for these sorts of highly non-linear systems without a detailed understanding of regional parameters. Therefore, better data related to regional scale are required to account for these outcomes. Furthermore, the quality of the regional data allows us a rare opportunity to generate data-driven statistical models to assess TB trends in Taiwan.

TB infection appears to persist throughout several years even in relatively small populations, possibly through recurring infections in adults. If individuals experiencing their first infection are the primary drivers of endemics, then demographic changes will have a strong influence on endemic time-series. Therefore, differences in population demographics and epidemiology of TB diseases, and, potentially, vaccine effectiveness, would need to be carefully considered when estimating the TB trends in local regions. Hsueh et al., [3] found that TB incidence in male patients was 2.2-fold higher than that in female patients. In this study, we found the similar results for male as higher prevalence group that can influence the TB trends in Taiwan (*p *< 0.001). Thus we thought that gender differences could be a factor accounting for the TB incidence.

We used AIC and RMSE to assess the model fitness in this study. The RMSE is a measure of the differences between the original datasets and predictive values by optimal model. The RMSE can also measure the average magnitude of the error by quadratic scoring function. The differences were being aggregated into a single measure to test the model predictive power. The AIC is a measure of the relative goodness of fit for statistical model. It is often used to describe the trade-off between bias and variance in model construction, or loosely speaking between accuracy and complexity of the model. AIC is an assessment for model selection not only reflects goodness of fit, but also includes a criterion that is an increasing function of the number of estimated parameters. Based on AIC method, our result found that Hwalien County had more optimal fitted models than Taitung County.

Our Poisson regression analysis indicated that the performance of RMSE did not obviously improve the predictability of TB trends in Taitung County by adding other predictors as age and gender. This revealed that these factors had only weak effects on the TB incidence in regional scale. Therefore, the age and gender show significant influence on TB trends in Hwalien County than in Taitung County.

### Limitations and implications

There are some limitations in our analyses. First, our database is limited to regional scale for which sufficient records were accessible to determine more significant predictors. A large gap is the age- and gender-specific TB cases in local scale, where data remain scarce. Likewise, we did not consider subpopulation effects, and had limited TB burden and trends data before 2004.

Second, our results are based on relatively coarse TB data provided by limited sources. The assessment of regional trends in incidence requires judgments about the reliability of case notifications reported by individual regional counties [22]. With these limitations, the results and forecasts present herein should be interpreted with caution.

Last, we analyzed only a subset of mechanisms that may shape TB trends regionally and nationally. We did not consider the factors such as numbers of alcoholics, diabetics, HIV, nature resistance-associated macrophage protein 1 (NRAMP 1), smokers, and military and inmate subpopulations, that have proven critical to TB trends variability [3,15,23-28]. Thus, those factors may be incorporated into the TB trends models to improve the predictability but they could have important dynamic consequences, which are worth exploring in future research. Generalized regression models rooted in regional data are important for providing clear recommendations for control strategies.

Although it was parameterized on the basis of observations from 2004-2007, the proposed Poisson regression model predicts the qualitative patterns of TB incidence rate at the regional and national scale in 2008. Assessing the impact of potential predictors on TB trends in Taiwan cannot neglect the self-intrinsic errors of missing variables for the selected Poisson regression model. This study suggests that more detailed surveillance data could well explain the peak values rather than missing information. Therefore, seeking novel mechanisms and providing both biological plausibility and epidemiological evidence for existing theories are still needed in future studies [22,29]. Assessment of TB trends in eastern Taiwan presents an important opportunity to understand the time-series dynamics and control of TB infections, given that this is the typical host demography in regions where these infections remain major public health problems.

## Conclusions

We found that in Taiwan the average TB incidence was 68 per 100,000 population with a mortality rate of 0.036 person^-1 ^yr^-1 ^in the period 2004-2008. The highest TB incidence rate was found in eastern Taiwan (116 per 100,000 population) with the largest proportion of TB relapse cases. We found that seasonality, aborigines, gender, and age had a consistent and dominant role in constructing TB incidence patterns in Taiwan, whereas these mechanisms were inconsistently supported for TB trends in regional scale. We also found that gender, time trend, and 2-month lag maximum temperature showed stronger association with TB trends in aboriginal subpopulations. The proposed Poisson regression model is capable of forecasting the patterns of TB incidence at the regional and national scales based on the previous data information. We anticipated that our Taiwan-based analysis can be extended to the context of developing countries, where *M. tuberculosis *remains a substantial cause of expected morbidity and mortality.

## Competing interests

The authors declare that they have no competing interests.

## Authors' contributions

CML contributed research concept and manuscript writing. NHH performed research concept design and tables/figures fabricating. TLH performed the statistical analysis and tables/figures fabricating. YHC and YJL associated the data collection and contributed to the data interpreting. CPC: solved the critical question in technique and provided valuable suggestion. SCC and MPL provided valuable suggestion and contributed the mainly discussion. All authors read and approved the final manuscript.

## Funding

No external sources of funding were received for this research

## Pre-publication history

The pre-publication history for this paper can be accessed here:

http://www.biomedcentral.com/1471-2458/12/29/prepub
